# Diet Shift May Trigger LuxS/AI-2 Quorum Sensing in Rumen Bacteria

**DOI:** 10.3390/bioengineering9080379

**Published:** 2022-08-10

**Authors:** Xiao Wei, Tanghui Long, Yanjiao Li, Kehui Ouyang, Qinghua Qiu

**Affiliations:** Jiangxi Province Key Laboratory of Animal Nutrition, Animal Nutrition and Feed Safety Innovation Team, College of Animal Science and Technology, Jiangxi Agricultural University, Nanchang 330045, China

**Keywords:** diet shift, LuxS/AI-2 quorum sensing, rumen bacteria community, rumen fermentation characteristic

## Abstract

Recent studies have revealed that LuxS/AI-2 quorum sensing (QS) is the most universal cell-to-cell communication in rumen bacteria; however, it remains unknown how they respond to nutritional stress from a diet shift. This study aimed to explore whether a diet shift could trigger rumen bacterial LuxS/AI-2 QS and its influences on rumen fermentation characteristics and bacterial community diversity and composition. A total of fifteen Hu sheep were selected to undergo a pre-shift diet (Pre, concentrate to forage ratio 75:25) for one month and then abruptly switch to a post-shift diet (Post, concentrate to forage ratio 49:51). Results showed that the serum cortisol and immunoglobulin G concentrations were higher in Post than in Pre (*p* < 0.05). The microbial density, AI-2 concentration, biofilm formation, and the gene expression of *ftsH* were higher in Post when compared with Pre (*p* < 0.05), whilst the gene expression of *luxS* tended to be lower in Post (*p* = 0.054). The molar concentration of valerate and fermentation efficiency decreased after the diet shift, while the acetate to propionate ratio and the molar proportion of butyrate were higher in Post compared to Pre (*p* < 0.05). Moreover, the diet shift increased the richness of ruminal bacteria and the relative abundances of *Roseburia*, *Prevotellaceae UCG-001*, and *Lachnospira*, and decreased the relative abundances of *Prevotella*, *Megasphaera*, and *Dialister* (*p* < 0.05). A difference in trends was also observed in an analysis of similarity (R = 0.1208 and *p* = 0.064). This study suggests that a diet shift could trigger rumen bacterial LuxS/AI-2 QS by altering microbial density, AI-2 concentration, biofilm formation, and related gene expression, as well as affect the rumen fermentation pattern and bacterial community diversity and composition. This study may provide insight into a potential strategy for relieving nutritional stress via regulating bacterial communication.

## 1. Introduction

The complex community of rumen microbiota, including bacteria, protozoa, fungi, and archaea, contributes substantially to the powerful ability of ruminants in digesting plant materials into exploitable nutrients [1]. Rumen bacteria have been regarded as the most diverse microbial community and have been studied extensively over the past decades [2,3,4], especially when next-generation sequencing technologies coupled with “omics” approaches became available and popular. These studies mainly focused on the interactions of diet–microbe, host–microbe, and environment–microbe [1,5,6]. However, ruminal bacteria are generally cooperative in the microbial fermentation of carbohydrates, with bacterial species contributing uniquely to maintaining normal and ecological balance [1,7]. Therefore, a deep understanding of the bacteria–bacteria network is urgently required to generate potential strategies for improving the productivity of ruminants.

Quorum sensing (QS) is a cell-to-cell communication mediated by signal molecules to regulate their phenotypes, such as biofilm formation, motility, and virulence [8,9]. The signal molecules include acyl-homoserine lactones (AHLs), autoinducer peptides (AIPs), and autoinducer-2 (AI-2), which are primarily secreted by gram-negative bacteria, gram-positive bacteria, and both gram-positive and gram-negative bacteria, respectively [10]. Moreover, the signal molecule AI-2 was proposed to be the universal language for interspecies communication [9]. Once the concentration of signal molecules secreted by bacteria reaches its threshold, other bacteria will sense the change and facilitate cooperative defense against an unfavorable environment via altering gene expression and phenotype [10]. Therefore, the QS system is actually a self-defense strategy to enhance bacterial adaptability and survival to the various habitat. Numerous studies have reported on bacteria-to-bacteria communication from a single species (e.g., *Escherichia coli*, *Salmonella typhimurium*, and *Vibrio harveyi*) of non-ruminant origin [11,12,13]; however, these communications in the rumen microbiome are limited. Since Erickson et al. [14] detected AHLs in ruminal contents, many reports have revealed the existence of AHLs in rumen bacteria [12,15,16]. Data from expression levels in rumen metatranscriptome datasets showed that LuxS/AI-2 QS was the most abundant in the rumen, and the genus of *Prevotella* expressed the highest LuxS synthase [17]. Similar data from rumen microbial metagenomics also revealed that AI-2-mediated QS system may participate in biofilm formation and fiber degradation [8]. However, Ran et al. [10] detected the presence of the gene *luxS*, but not the AI-2 signaling molecules, both in vivo and in vitro, probably due to the limitations of detection techniques or pre-adapted diets. Environmental changes, such as initial pH, carbon sources, and glucose concentration, were reported to stimulate AI-2 production and *luxS* transcription [18]. These results indicated that bacterial LuxS/AI-2 QS may be opportunistic in rumens of ruminants.

A modern ruminant production system generally involves several growth or finishing stages, and each stage requires a unique nutritional feeding strategy to meet its requirement for maintenance and production [19]. A diet shift is the most popular manner in which to adjust a feeding strategy, and a rapid shift leads to a perturbed microbial community, reduced performance, and poor host immune response [20,21]. Many reports have revealed the strong associations between improved host health and production and stable ruminal microbiomes [20,22]. Therefore, less time to achieve the stability of microbiomes will improve host productivity, and the regulation of ruminal bacteria communications, such as QS, may be a potential strategy for shortening the adaptation time before stability.

This study attempted to uncover the possibility of a diet shift on the triggering of LuxS/AI-2 QS, and provide further insight into a new strategy for relieving stress upon a diet shift. We hypothesized that a diet shift would trigger the LuxS/AI-2 QS of rumen bacteria, and that cooperative behavior would be activated to fight against adversity due to the dietary shift.

## 2. Materials and Methods

### 2.1. Animals and Experimental Design

This experiment was conducted at the test site of Jiangxi Agricultural University. All animal care and welfare guidelines were strictly followed from the Committee for the Care and Use of Experimental Animals at Jiangxi Agricultural University (JXAULL-2021036). A total of fifteen Hu sheep, with average body weight of 16.03 ± 0.55 kg and age of 3.09 ± 0.16 months, were selected to undergo a diet shift. The pre-shifted diet (Pre, concentrate to forage ratio 75:25) was the routine diet for feeding these sheep for one month, and the post-shifted diet (Post, concentrate to forage ratio 49:51) was another popular ration for growing sheep in China according to the Chinese Feeding Standard of Sheep (NY/T816-2004). Both Pre and Post diets were designed with a similar metabolic energy (ME) to crude protein (CP) ratio of 0.068 MJ/g to avoid extra effects due to energy or protein balance. The ingredient and chemical composition of these two diets are shown in Table 1. All animals were fed twice a day at 7:30 and 17:30, and approximately 10% feed residues were kept for them to allow ad libitum. Round-the-clock fresh water was provided for the tested sheep and they suffered from no other stress except for the diet shift.

### 2.2. Sample Collection

Samples from each sheep were collected the day before the diet shift and the day after the diet shift. Blood samples were collected from jugular vein by means of vacuum vasculature without anticoagulant before morning feeding, and the serum was obtained by centrifuging blood samples at the speed of 3000 rpm/min for 20 min. Rumen contents, including both liquid and solid fractions, were collected before morning feeding using the esophageal tubing method as described by Paz et al. [23]. The rumen pH was determined after the rumen contents were removed using a portable pH meter (Testo 206, Testo AG, Schwarzwald, Germany). The rumen fluid was then obtained by filtering rumen contents through four layers of gauze, and these samples were stored at −80 °C and used for both DNA and RNA extraction and for rumen fermentation characteristics determination.

### 2.3. RNA Extraction, LuxS/AI-2 Quorum Sensing, and Biofilm Formation Assay

The method of TRIzol extraction after bead beating was taken to extract the RNA of rumen fluid as Kang et al. [24] described, as they reported that this method yielded higher quantity and quality of rumen bacterial RNA when compared with the traditional method using phenol/chloroform. The concentration and purity of extracted RNA were determined by an ultrafine spectrophotometer (NanoDrop 2000, Thermo Fisher Scientific, Waltham, MA, USA), and all RNA samples were diluted to 200 ng/µL to perform the following reverse transcription to obtain cDNA. The genus of *Prevotella* was selected as the target bacteria, as a recent metatranscriptome dataset analysis revealed that *Prevotella* expressed the highest level of LuxS synthase [17]. The *luxS* and *ftsH* of *Prevotella* were the target genes for LuxS/AI-2 QS and biofilm formation, respectively, and the 16S rRNA was used as the reference gene. The detailed primer information is listed in Table 2. A real-time quantitative PCR was performed in a CFX96 Touch Real-Time PCR Detection System (Bio-Rad Laboratories, Inc., Hercules, CA, USA), and each sample was conducted in triplicate. The relative expressions of target genes were calculated by the method of $2^{{-\Delta \Delta C}_{T}}$ [25].

Ruminal bacteria density was expressed as the absorbance at the wavelength of 595 nm (OD_595_) after centrifuging at 10,000× *g* for 5 min [26]. The AI-2 concentration was quantified with the colorimetric method developed by Wattanavanitchakorn et al. [27]. In brief, the supernatant of rumen fluid was obtained by centrifugation at 12,000× *g* for 10 min and was then filtered through 0.45 µm and 0.22 µm membrane in sequence. The filtered supernatant was mixed evenly with Fe (III)—1, 10-phenanthroline reagent in equal volume, and then the mixture was left to stand for 1 min to allow a complete chromogenic reaction. Absorbance of colored [(o-phen)_3_ Fe(II)]SO_4_ ferroin complex was detected within 3 min at a wavelength of 510 nm using an absorbance microplate reader (SpectraMax 190, Molecular Devices, San Jose, CA, USA). The standard curve was drawn using ascorbic acid with the same procedures as mentioned above, with 20 µM ascorbic acid as the positive control and the deionized distilled water as the negative control. Biofilm formation assay was performed with the crystal violet staining method as described in Gu et al. [26] wherein methanol, crystal violet, and ethanol were used as fixative, stain, and decolouration, respectively. The extracellular polymeric substances were extracted following the method described by Gu et al. [26]. The amount of exopolysaccharide was determined using the phenol-sulfuric acid method with glucose as the standard [28], and the extracellular protein was measured by the Lowry method [29].

### 2.4. Serum Indicators and Rumen Volatile Fatty Acids Determination

Serum parameters included glucose, cortisol, and immunoglobulin G (Ig G), which are indicators of energy metabolism, stress status, and immunocompetence, respectively. The concentration of glucose was measured using the glucose oxidase method by an automatic biochemical analyzer (HITACHI7170, Hitachi Limited, Kokyo, Japan). The serum cortisol and Ig G concentrations were detected by their corresponding commercial kits manufactured by the Beijing Sinouk Institute of Biological Technology (Beijing, China). Volatile fatty acids (VFA) determined in this study included acetate, propionate, isobutyrate, butyrate, isovalerate, and valerate, and the sum of isobutyrate, isovalerate, and valerate was defined as branched chain volatile fatty acids (BCVFA). The identification and concentration of each individual VFA referred to the relative retention time and peak area of the standard curve, respectively. Both the standard curve and sample determination were performed by a gas chromatograph (GC-2014 Shimadzu Corporation, Kyoto, Japan) with nitrogen as the carrier gas, and the injection volume and injector temperature were kept at 0.4 µL and 220 °C, respectively. The oven procedure was the same as Qiu et al. [30]. Rumen fermentation patterns were indicated by the acetate to propionate ratio, non-glucogenic to glucogenic acids ratio (NGR), and fermentation efficiency. NGR was calculated as (C2 + 2 × C4 + C5)/(C3 + C5) and fermentation efficiency was expressed as (0.622 × C2 + 1.092 × C3 + 1.56 × C4)/(C2 + C3 + 2 × C4), where C2, C3, C4, and C5 indicate acetate, propionate, butyrate, and valerate, respectively [31].

### 2.5. DNA Extraction, Sequencing, and Data Analysis

A total of sixteen rumen fluid samples, with eight before the diet shift (homogeneously mixing the first sample (S1) and the last sample (S15), the second sample (S2) and the penult sample (S14), …, S7 and S9, single S8) and eight after diet shift (the same mixture method as the former), were extracted using a bacterial DNA Kit (OMEGA, Omega Bio-Tek, Norcross, GA, USA) with special caution taken to follow the manufacture’s instruction. The purity of extracted DNA was checked on 1% agarose gels, and quality and concentration were evaluated by a Qubit 2.0 Fluorometer (Life Technologies, Carlsbad, CA, USA). The bacterial V3 to V4 regions were amplified with barcoded primers as follows: 338F (5′-ACTCCTACGGGAGGCAGCAG-3′) and 806R (5′-GGACTACNNGGGTATCTAAT-3′). The PCR reaction system and amplification program were the same as Qiu et al. [32] described. Each sample was performed in triplicate, and PCR products were checked on 1% agarose gels and purified using an Agencourt AMPure XP Kit (Beckman, Brea, CA, USA). High throughput sequencing was performed on Illumina Miseq PE300 platform by Allwegene Gene Technology Co., Ltd. (Nanjing, China), and paired-end reads were generated for the subsequent data analysis.

Sequencing data were analyzed using QIIME 2 (https://qiime2.org/, accessed on 5 May 2022, [33]). The raw data were removed if they met one of the following criteria: sequences length less than 250 bp or greater than 500 bp; evaluated quality score below 20; and containing ambiguous bases or not exactly matching to primer sequences and barcode. The filtered data were merged into tags by paired-end reAd mergeR (PEAR, v0.9.6, [34]) software, where the minimum overlap and the mismatch rate were set to 10 bp and 0.10, respectively. Qualified tags were denoised into amplicon sequence variants (ASVs) by means of the Deblur algorithm of QIIME 2. Taxonomic classifications for each ASV were obtained using the Ribosomal Database Project (RDP) Classifier tool with a confidence threshold of 0.70, wherein the database of bacterial SILVA 132 was taken to be assigned against. Alpha diversity metrics, including Chao 1, observed species, phylogenetic diversity (PD) whole tree, Shannon index, and Simpson index, were calculated to evaluate the richness and evenness of each sample, and were finished by QIIME 2 based on ASV information. Principal coordinates analysis (PCoA) and non-metric multidimensional scaling (NMDS) were performed to show the differences or similarities between Pre and Post based on Bray–Curtis distances. Analysis of similarity (ANOSIM) was conducted to reveal the similarities between Pre and Post using the vegan package in the R software.

### 2.6. Statistical Analysis

After the confirmation of normal distribution using the Shapiro–Wilk test, all data involved in this study were analyzed using the paired sample t-test of SPSS (version 20, IBM Corporation, Armonk, NY, USA). Significant difference was declared at 0.05 (*p* < 0.05), and a trend was introduced if *p* value was between 0.05 and 0.10 (0.05 ≤ *p* < 0.10).

## 3. Results

### 3.1. Serum Biochemical, Immune, and Hormonal Indicators

**Figure 1:**
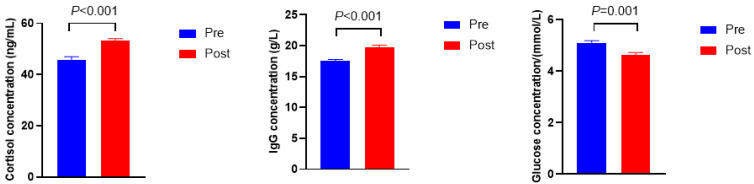
Serum biochemical, immune, and hormonal indicators before (Pre) and after (Post) diet shift.

As shown in Figure 1, the cortisol and immunoglobulin G (Ig G) concentrations were higher in Post when compared with Pre (*p* < 0.001), whereas the concentration of glucose was lower in Post than in Pre (*p* = 0.001).

### 3.2. AI-2 Concentration and luxS Gene Expression

**Figure 2:**
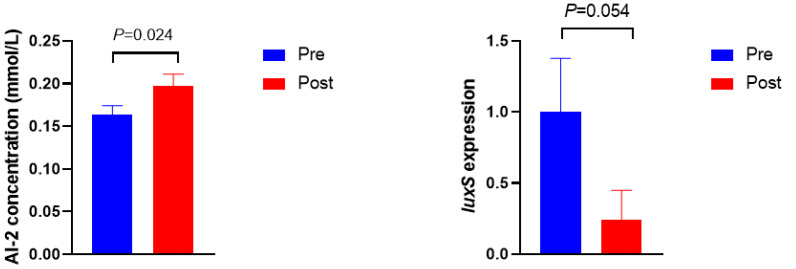
AI-2 concentration and *luxS* gene expression before (Pre) and after (Post) diet shift.

The AI-2 activity and *luxS* gene expression are shown in Figure 2. The concentration of AI-2 was higher in Post than in Pre (*p* = 0.024). Moreover, the expression of the *luxS* gene tended to be lower in Post when compared with Pre (*p* = 0.054).

### 3.3. Microbial Density, Biofilm Formation, ftsH Gene Expression, and Extracellular Polymeric Substances Composition

**Figure 3:**
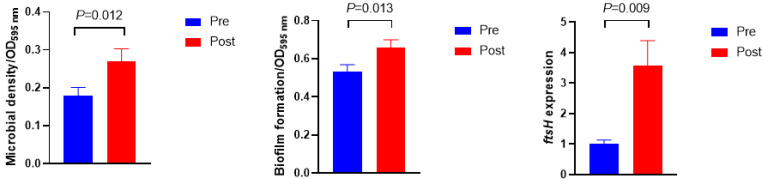
Microbial density, biofilm formation, and its related gene expression before (Pre) and after (Post) diet shift.

**Figure 4:**
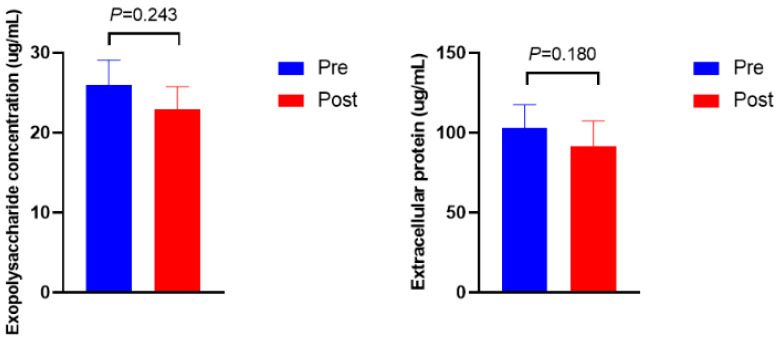
Exopolysaccharide and protein concentrations of extracellular polymeric substances before (Pre) and after (Post) diet shift.

Figure 3 shows the microbial density, biofilm formation, and *ftsH* gene expression. The microbial density and biofilm formation were higher in Post than in Pre (*p* = 0.012 and *p* = 0.013, respectively). The expression of the *ftsH* gene in Post was 3.5-fold higher than in Pre (*p* = 0.009). No significant differences were observed in the exopolysaccharide and protein concentrations of extracellular polymeric substances (Figure 4, *p* > 0.10).

### 3.4. Rumen Fermentation Characteristics

Rumen fermentation characteristics before and after the diet shift are presented in Table 3. The molar concentration of valerate was lower in Post than in Pre (*p* = 0.049). The acetate to propionate ratio and NGR were found to be higher in Post, whereas fermentation efficiency decreased after the diet shift (*p* < 0.05). The molar proportions of propionate and valerate were lower in Post compared to Pre, whilst the former showed an increased butyrate proportion (*p* < 0.05). The molar concentration and proportion of BCVFA tended to be higher in Pre than in Post (*p* = 0.092 and *p* = 0.072, respectively).

### 3.5. Rumen Bacterial Diversity and Community Structure

The alpha diversity metrics before and after the diet shift are shown in Table 4. Chao 1, observed species, and PD whole tree were higher in Post when compared with Pre (*p* < 0.05). No significant differences were observed in the Shannon index and Simpson index between Pre and Post (*p* > 0.10).

As shown in Table 5, eight phyla were found with a relative abundance greater than 0.10%. The abundance of *Actinobacteriota* in Pre tended to be higher than in Post (*p* = 0.059), whereas other phyla showed no differences between Pre and Post (*p* > 0.10).

A total of nineteen genera were observed with a relative abundance above 0.50% (Table 6). The relative abundance of *Prevotella*, *Megasphaera*, and *Dialister* were higher in Pre than in Post (*p* < 0.05), whereas *Roseburia*, *Prevotellaceae UCG-001*, and *Lachnospira* abundances were higher in Post when compared with Pre (*p* < 0.05). The relative abundance of *Acetitomaculum* in Pre tended to be higher compared to Post (*p* = 0.072).

**Figure 5:**
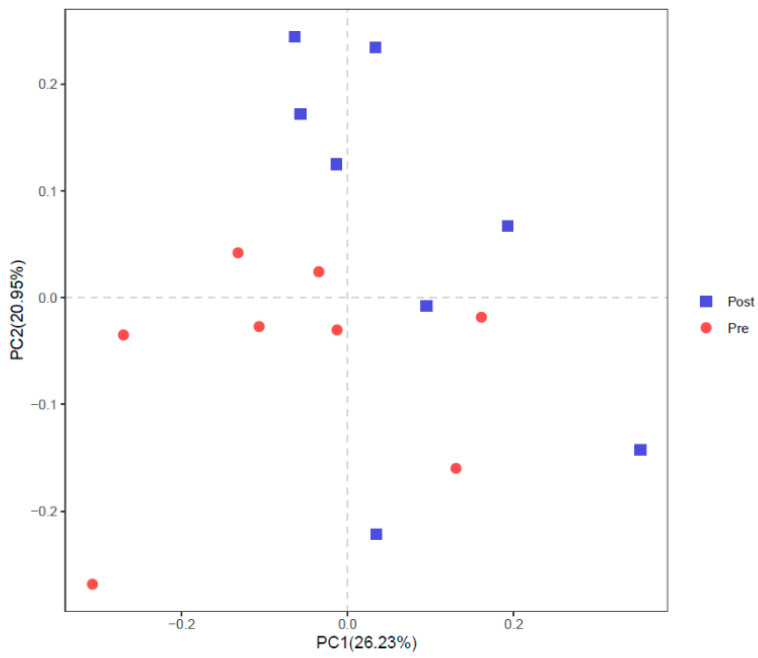
Principal coordinates analysis (PCoA) of rumen bacterial community before (Pre) and after (Post) diet shift.

**Figure 6:**
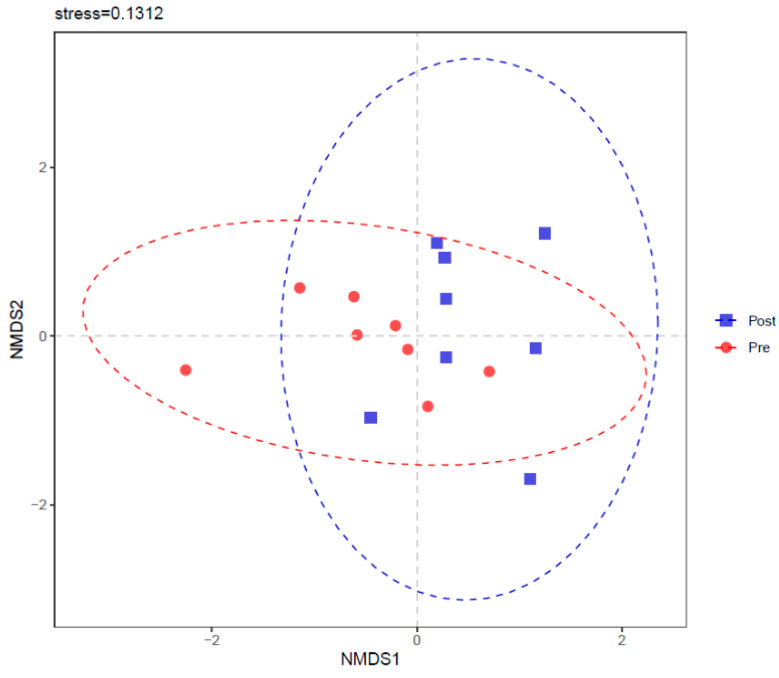
Non-metric multidimensional scaling (NMDS) of rumen bacterial community before (Pre) and after (Post) diet shift.

Part intersections were observed in PCoA (Figure 5) and NMDS (Figure 6) between Pre and Post. ANOSIM also showed a trend of difference between Pre and Post with R = 0.1208 and *p* = 0.064.

## 4. Discussion

When the dietary concentrate to forage ratio shifted abruptly from 75:25 to 49:51, serum physicochemical characteristics changed accordingly. As a sensitive hormonal indicator of stress, the level of cortisol increases remarkably by activating the hypothalamic–pituitary–adrenocortical (HPA) axis when animals and humans suffer from stressors [35]. A higher concentration of cortisol indicated that the diet shift caused stress successfully. It is widely known that, once stress occurs, the body initiates an immune response to withstand adversity stress. As the main immunoglobulin class among five distinct immunoglobulins, Ig G plays a decisive role in maintaining normal life [36]. This study found that the concentration of Ig G increased after the diet shift, indicating that a defensive response was activated to fight against nutritional stress. Glucose concentration is a critical indicator for energy metabolism, and its value increased with greater intake of digestible energy or more concentrate [37,38]. The serum glucose before the diet shift showed higher levels, which could be attributed to the higher density of energy in Pre when compared with the energy density in Post. Another possible explanation for the lower concentration of glucose in Post would be the fact that glucose could be synthesized from rumen propionate via hepatic gluconeogenesis [39], because numerically higher molar concentration and higher molar proportion of propionate were observed in Pre.

As the primary QS in rumen bacteria revealed by both metagenomics and metatranscriptome dataset analysis, LuxS/AI-2 QS plays a vital role in the interspecies communication of rumen bacteria [8,17]. AI-2 is the core and featured signaling molecule in LuxS/AI-2 QS system, and its concentration could be perceived by bacteria to coordinate collective behavior, such as cell density and biofilm formation [8,9]. In the current study, the concentration of AI-2 increased after the diet shift; meanwhile, increased microbial density and more biofilm formation were observed, indicating that bacteria had exhibited collective behaviors when the signaling molecule concentration reached its threshold to defend against the abrupt diet shift. These phenotype variations were regulated by their corresponding gene expressions, namely *luxS* for AI-2 synthesis and *ftsH* for biofilm formation. The gene *luxS* encodes autoinducer 2 synthase (LuxS), which is responsible for the synthesis of AI-2 [8,17]. The sequences of the *luxS* gene were found to be abundant in *Prevotella*, *Butyrivibrio*, *Ruminococcus*, *Pseudobutyrivibrio*, and *Eubacterium* [8,17]. In this study, a decline in the trend of *luxS* gene expression was observed in *Prevotella* after the diet shift even with a higher AI-2 concentration, probably due to the uncharacterized relationships between diverse *luxS*-containing bacteria and AI-2 secretion [26]. Another possible explanation for the inconsistent results is that they may be attributed to the lower serum glucose in Post, because a previous study found that the level of *luxS*-mRNA decreased when deficient glucose was supplied to *Streptococcus bovis* [40]. Biofilm is a complex three-dimensional structure of bacterial aggregates, mainly composed of polysaccharides, proteins, nucleic acids, and lipids [41,42]. Its existence proved more resistant to stress when compared with its planktonic counterpart, and mixed-species biofilm exhibited more resistance to environmental stress than single-species biofilm [42,43]. The gene *ftsH* was proposed to be involved in biofilm formation to protect against stress, and its mutant strain showed a reduced biofilm formation capacity [44]. Higher gene expression of *ftsH*, as well as more biofilm formation, was observed in Post when compared with Pre, indicating that the diet shift stimulated more biofilm formation via up-regulating the gene expression of *ftsH*. However, extracellular polysaccharide and protein concentrations did not show a significant difference in rumen fluid between Pre and Post, which is probably due to the distinct development, structure, and function of biofilm between mixed species and single species [43]. This study revealed a positive association between AI-2 concentration and biofilm formation, which is in line with previous reports [26,45]. Moreover, the current results also revealed that the *ftsH* gene was more sensitive to the diet shift than to the *luxS* gene, indicating that biofilm formation may be prioritized over AI-2 synthesis when undergoing a diet shift. These results suggest that the diet shift triggered rumen bacterial LuxS/AI-2 QS, and ruminal bacteria cooperated together to defend against the diet shift by improving microbial density, AI-2 synthesis, and biofilm formation.

The diet shift altered ruminal fermentation patterns and efficiency, but did not affect the absolute concentrations of VFA and individual VFA, except for valerate. Generally, VFA is considered as the primary energy utilization form of ruminants, and a higher NGR indicates a lower FE of dietary energy from carbohydrates to VFA [31]. In this study, higher NGR and lower FE were observed after the diet shift, indicating that the diet shift impeded rumen fermentation. Liu et al. [46] reported that an increase in dietary concentrate feeding yields a higher concentration of valerate; similar results were observed for the valerate concentration of Post regarding both concentration and proportion, probably due to the reduction in dietary concentrates in Post. It is easy to explain why Post showed a decreased proportion of propionate and a higher acetate to propionate ratio because of the well-established theory that structural carbohydrates produce more acetate and less propionate when compared with non-structural carbohydrates [47]. A higher proportion of butyrate was found in Post, which is inconsistent with previous reports that a high-density diet produced more butyrate [32]; this is probably explained by the fact that the VFA of Pre decreased faster due to its high-grain composition [48]. Branched-chain volatile fatty acids, including isobuyrate, valerate, and isovalerate, are the main products of crude protein degradation and are commonly used to monitor protein fermentation [49]. Therefore, it is reasonable to expect the increments in the trend of concentration and proportion of BCVFA in Pre when compared with Post due to differences in dietary crude protein (16.14% vs. 14.12%).

The diet shift altered the rumen bacterial richness, which was indicated by Chao 1 and observed species. A previous study revealed that a high-density diet decreased ruminal bacteria richness and evenness [32], and the current study found similar results, since the dietary energy and protein in Pre were higher than in Post. Zhao et al. [50] reported higher Actinobacteriota abundance when the concentration of oxygen decreased, probably explaining the trending decline of this phylum in Post because more roughage could carry more oxygen into the rumen [51]. The genus *Prevotella* is involved in fermenting starch and degrading protein [52], which partly explains its higher abundance in Pre due to its higher dietary protein and energy density. However, as a genus belonging to the family Prevotellaceae, *Prevotellaceae UCG-001* showed opposing results to *Prevotella*, indicating not all genera in the same family share similar responses to dietary change. Scott et al. [53] reported that *Roseburia* was identified as a producer of butyrate, and the current results revealed a higher abundance of this genus in Post, which corresponded well to the higher molar proportion of buyrate in Post. *Megasphaera* is a predominant gram-negative commonly found in the rumens of cattle fed high-grain diets [54], and its main function is to ferment lactate into acetate and propionate [55]. Therefore, it is expected that the higher abundance of *Megasphaera* was observed in Pre because of the high dietary concentrate to forage ratio. Similar to *Megasphaera*, *Dialister* possesses the capability of utilizing simple sugar [56], probably providing evidence for the higher abundance of this genus in Pre due to the higher content of non-structure carbohydrates. The family Lachnospiraceae is commonly isolated from the rumens of cattle fed high-fiber diets and plays a vital role in facilitating forage degradation [57], and Liu et al. [58] found higher abundances of genera in this family when goats were fed an all-forage diet. These findings explain the current higher abundance of *Lachnospira* in Post because sheep in this treatment received more fiber (Table 1).

## 5. Conclusions

Taken together, the diet shift increased the concentrations of serum cortisol and Ig G, rumen AI-2 concentration, microbial density, biofilm formation, and the gene expression of *ftsH*. The diet shift also affected the rumen fermentation pattern by increasing the acetate to propionate ratio, decreasing fermentation efficiency, and altering molar proportions of propionate, butyrate, and valerate. Moreover, the diet shift altered the richness of the rumen bacterial community and some of the bacteria at the level of phylum or genus. This study suggests that a diet shift could trigger rumen bacterial LuxS/AI-2 QS by altering microbial density, AI-2 concentration, biofilm formation, and related gene expression, as well as affect the rumen fermentation pattern and bacterial diversity and community composition. This study may provide insight into a potential strategy for relieving nutritional stress via regulating bacterial communication.

## Figures and Tables

**Table 1:** Ingredients and nutritional composition of pre- and post-shift diet.

Item	Pre-Shift Diet	Post-Shift Diet
Ingredients, % of dry matter (DM)		
Corn	36.20	20.00
Soybean meal	19.47	19.80
Wheat bran	13.24	3.49
Wheat straw	25.09	50.71
Calcium bicarbonate	0.50	0.50
Calcium hydrophosphate	0.50	0.50
Limestone	0.50	0.50
Salt	0.50	0.50
Premix ^1^	4.00	4.00
Total	100	100
Nutritional composition, g/kg of DM		
Metabolic energy (ME), MJ/kg	10.97	9.59
Crude protein (CP)	161.4	141.2
ME to CP ratio, MJ/g	0.068	0.068
Neutral detergent fiber (NDF)	308.6	428.0
Acid detergent fiber (ADF)	164.8	266.4

^1^ Premix provided the following per kg of DM: 1400 mg of Fe, 1200 mg of Zn, 250 mg of Cu, 900 mg of Mn, 100,000 IU of vitamin A, 27,000 IU of vitamin D3, and 800 IU of vitamin E.

**Table 2:** Primers designed for gene expressions of LuxS/AI-2 quorum sensing and biofilm formation.

Accession Number	Gene Name	Primer Sequence (5-3)	Product Size (bp)
SEW05678.1	16S rRNA-F	AGAGCCTGAACCAGCCAAGTAG	128
	16S rRNA-R	GAATTAGCCGGTCCTTATTCATACA	
	*luxS*-F	GGATGATGTAGTGTATGTCGGTCC	185
	*luxS*-R	GGAGGTCGTGGAGCAGATAGTT	
SHK82531.1	16S rRNA-F	TGCGTCTGATTAGGTAGTAGGCG	112
	16S rRNA-R	CGTAGGAGTTTGGACCGTGTCT	
	*ftsH-*F	AGATGTATGAGAAGGGTGGTGAGT	146
	*ftsH-*-R	TCCCTTGGGTATCTTACCTCCC	

**Table 3:** Rumen fermentation characteristics before (Pre) and after (Post) diet shift.

Item ^1^	Pre	Post	SEM ^2^	*p*-Value
pH value	6.94	6.95	0.07	0.821
Molar concentration (mM)				
Acetate	11.06	10.68	1.137	0.743
Propionate	5.89	4.94	0.673	0.180
Isobutyrate	0.11	0.10	0.022	0.448
Butyrate	1.26	1.52	0.171	0.153
Isovalerate	0.18	0.16	0.019	0.327
Valerate	0.56	0.47	0.044	0.049
Total volatile fatty acids	19.07	17.86	1.945	0.546
Branched-chain volatile fatty acids	0.86	0.72	0.073	0.092
Acetate to propionate ratio	1.98	2.28	0.114	0.017
NGR	2.29	2.73	0.117	0.002
Fermentation efficiency	0.78	0.77	0.005	0.022
Molar proportion (mol/100 mol)				
Acetate	58.62	60.17	1.137	0.194
Propionate	30.30	27.21	1.041	0.010
Isobutyrate	0.64	0.59	0.102	0.678
Butyrate	6.51	8.40	0.673	0.014
Isovalerate	0.99	0.94	0.056	0.406
Valerate	2.95	2.68	0.111	0.032
Branched-chain volatile fatty acids	4.57	4.22	0.183	0.072

^1^ Branched-chain volatile fatty acids are the sum of isobutyrate, valerate, and isovalerate; NGR, non-glucogenic to glucogenic acids ratio. ^2^ SEM, standard error of the mean.

**Table 4:** Rumen bacterial alpha diversity metrics before (Pre) and after (Post) diet shift.

Item	Pre	Post	SEM	*p*-Value
Chao 1	592.76	648.48	10.47	0.001
Observed species	506.13	560.74	9.41	0.001
PD whole tree ^1^	30.63	33.00	0.44	0.001
Shannon index	5.44	5.61	0.17	0.383
Simpson index	0.94	0.94	0.02	0.807

^1^ PD whole tree, phylogenetic diversity whole tree.

**Table 5:** Rumen bacterial community at the level of phylum before (Pre) and after (Post) diet shift.

Item	Pre	Post	SEM	*p*-Value
Bacteroidetes	51.46	46.40	2.89	0.124
Firmicutes	27.21	25.50	4.09	0.689
Proteobacteria	18.56	25.19	5.38	0.257
Cyanobacteria	0.74	1.07	0.26	0.237
Actinobacteriota	1.00	0.62	0.17	0.059
Fibrobacterota	0.53	0.64	0.46	0.822
Desulfobacterota	0.25	0.27	0.06	0.823
Spirochaetota	0.12	0.19	0.04	0.121

**Table 6:** Rumen bacterial community at the level of genus before (Pre) and after (Post) diet shift.

Item	Pre	Post	SEM	*p*-Value
*Prevotella*	49.72	42.77	2.79	0.042
*Succinivibrio*	15.50	21.95	5.15	0.251
*Roseburia*	1.42	5.64	1.75	0.047
*Succinivibrionaceae UCG-001*	2.78	2.70	0.81	0.922
*Selenomonas*	3.44	1.94	1.02	0.185
*Erysipelotrichaceae UCG-002*	4.00	0.55	2.90	0.274
*Megasphaera*	2.53	0.72	0.34	0.001
*Syntrophococcus*	1.22	1.28	0.17	0.719
*Acetitomaculum*	1.85	0.52	0.63	0.072
*Dialister*	1.46	0.91	0.16	0.011
*Oribacterium*	1.04	1.26	0.34	0.552
*Prevotellaceae UCG-001*	0.20	1.41	0.31	0.006
*Lachnospiraceae NK3A20 group*	0.94	0.58	0.37	0.348
*Acidaminococcus*	0.87	0.58	0.17	0.126
*Ruminococcus*	0.86	0.43	0.25	0.132
*Succiniclasticum*	0.49	0.80	0.18	0.123
*Fibrobacter*	0.53	0.64	0.46	0.822
*Olsenella*	0.69	0.47	0.12	0.119
*Lachnospira*	0.26	0.77	0.13	0.006

## Data Availability

The raw data involved in this study were deposited in the Sequence Read Archive (SRA) of NCBI with the accession number of PRJNA835689.
